# Bursting on the Scene: How Thalamic Neurons Grab Your Attention

**DOI:** 10.1371/journal.pbio.0040250

**Published:** 2006-06-13

**Authors:** Jami Milton Dantzker

Certain salient features in a visual scene grab our attention, such as a tiger emerging from a field of tall grass, after which we may spend more time taking in the details. Both ways of experiencing the world are automatic, and the neurons that make up the brain's visual system switch between these two modes continuously. But the exact neurons and mechanisms responsible are still a mystery to neuroscientists.

Based on their physiological properties and connectivity, neurons in the region of the brain known as the thalamus seem especially well-suited to responding dynamically to our visual world. The thalamus is considered the relay station for the brain, shuttling information between sensory neurons and the cerebral cortex. Thalamic neurons in the lateral geniculate nucleus (LGN) respond to visual scenes in two distinct modes: sustained and regular tonic responses and short, rapid bursts. Neuroscientists have long been curious about the role of these distinct firing modes in processing visual information, and whether certain features in a visual scene elicit them.

To better understand the function of bursts, Nicholas Lesica, Garrett Stanley, and colleagues investigate the interplay between external stimuli and intrinsic physiological membrane properties in modulating LGN burst responses. In a new study, they record LGN neuron activity in response to sequences of movies showing natural scenes that vary in luminance, then mathematically simulate the neurons' response properties under different intrinsic physiological states, and with or without the burst mechanism.

The researchers incorporated several well-established properties of LGN neurons into their model. For example, bursting is known to depend on a fundamental physiological feature, a neuron's membrane potential. The membrane potential refers to the electrical potential, or voltage, across the membrane resulting from a balance of negatively and positively charged ions, such as calcium. When neurons are at rest, they sit at a negative, or lower, potential; but when they are activated by a stimulus or other neurons, the potential becomes more positive, or higher. Once a certain positive voltage threshold is reached, the neuron fires an action potential. Lesica et al. designed “integrate-and-fire” model neurons with exactly this threshold property.

Similarly, bursting is also based on a threshold mechanism. Bursts are mediated by “T-type” calcium channels (T channels) that pass positively charged calcium ions into the cell; they become inactive at relatively higher membrane potentials. To model burst firing due to T channels, Lesica et al. combined a low-threshold, voltage-dependent current (channels open at relatively low voltage membrane potentials). The model also mimicked the spatial properties that activate LGN neurons. The model allowed the researchers to tweak the membrane potential of their simulated LGN neurons during the movies. And to understand how the bursting affected a neuron's response, they removed the burst-firing mechanism altogether.

By systematically changing the membrane potential, the researchers observed a strong relationship between resting potential, T channel threshold potential, and burst firing. The percentage of bursts in the LGN response was greatest when the resting potential was much more negative than the threshold potential. To characterize how luminance changes in the stimulus and membrane potential interact to evoke bursts, they analyzed the luminance features that preceded bursts for several hundred milliseconds. They found that when the resting potential was lower than the T channel threshold potential, an excitatory stimulus alone could trigger a burst. In contrast, at higher resting potentials, a prolonged inhibitory stimulus was required (to de-inactivate the T channels) before an excitatory stimulus could evoke a burst. In either case, they found that bursts mediated an enhanced detection of the stimulus, similar to a “wake-up call” to pay attention to the stimulus. When the researchers removed the bursting mechanism in their model neurons, they found that the tonic mode of firing was not able to induce the same level of enhanced responses. They suggest instead that tonic firing may be important for conveying the details of a stimulus.

The analysis supports a role for the thalamus in directing attention, and as the researchers explain, reveals that bursting could be used as a reliable signal to direct attention-related resources toward a behaviorally relevant area of the visual field. The mathematical simulations allowed Lesica et al. to test the role of bursting in a way that is not currently feasible with real neurons. And their findings pave the way for future experimental studies showing how LGN burst and tonic responses faithfully represent the dynamic visual world in behaving animals.

## 

**Figure:**
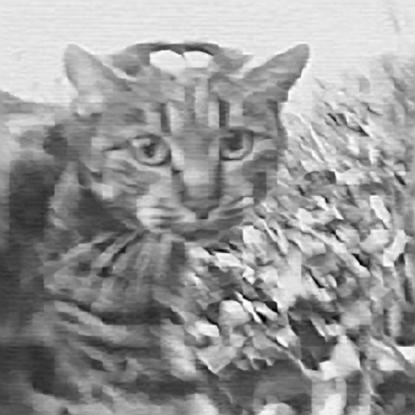
This image shows a frame from the natural scene movies used in Lesica et al. To obtain the scenes, researchers in the Koenig group at ETH in Zurich mounted a cat with a camera; in this particular movie segment, the cat carrying the camera encountered another cat.

